# Combined strategies on the treatment of cerebellar arteriovenous malformation

**DOI:** 10.3171/2020.10.FOCVID2058

**Published:** 2021-01-01

**Authors:** Marcos Dellaretti, Diego da Silveira, Tancredo Alcântara Ferreira Junior

**Affiliations:** 1 Neurosurgery and Neurology Department, Santa Casa de Belo Horizonte, and; 2 Federal University of Minas Gerais, Belo Horizonte, Minas Gerais, Brazil

**Keywords:** cerebellar arteriovenous malformation, unruptured AVM, microsurgery, embolization

## Abstract

Cerebellar arteriovenous malformations (AVMs) comprise 10%–15% of all intracranial AVMs and have a higher risk for morbidity and mortality than supratentorial AVMs. Patients with cerebellar AVMs present with hemorrhage more often than patients with cerebral AVMs, justifying an interventional treatment. Patient outcome can be predicted with specific grade systems, guiding vascular neurosurgeons in decision-making. The authors present the case of a 42-year-old man incidentally diagnosed with an unruptured cerebellar inferior vermian AVM, which was managed through a combined strategy of preoperative endovascular embolization of the main arterial feeders followed by microsurgical resection via midline suboccipital craniotomy, with a favorable outcome.

The video can be found here: https://youtu.be/3WESejZbk90

## Transcript

**0:26 Clinical Presentation.** The patient was 42 years old with headache for 3 months. He had a Glasgow Coma Scale score of 15 and no focal neurological deficits.

**0:36 Imaging.** MRI showed an inferior vermian AVM.

**0:39** Left vertebral angiography shows the cerebellar AVM’s main arterial supply was the left PICA, and the basilar artery did not fill by the left vertebral artery due to steal phenomena. The size of the AVM was 2 cm.

The blue arrows show that the left PICA had three flow aneurysms. In the figure on the right, we see the arterial supply of the AVM. It was done from the branches of the telovelotonsillar segment of the PICA.

Regarding venous drainage, at the red arrow, we see a venous aneurysm in the vein of Galen; at the brown arrow, the basal veins of Rosenthal; at the green arrow, the straight sinus. The yellow arrow shows the main drainage vein of the AVM.

**1:32** This drawing (Supplemental Fig. 1) shows the AVM. The left PICA is shown transparent inside of the cerebellum and provides the final branches of the telovelotonsillar segment for supplying the AVM. There is a small cortical branch from the left vertebral artery that supplies the AVM. The AVM is shown transparently. Venous drainage is primarily performed to the inferior vermian vein. The vein is shown in purple here because it was an arterialized vein. The venous aneurysm is shown here near the vein of Galen.

**2:16** This was an infratentorial AVM with deep venous drainage, which are risk factors for rupture.^1–3^ Hemorrhage due to prenidal aneurysm also occurs more frequently in cerebellar AVMs than in cerebral AVMs.

The Spetzler and Martin AVM classification indicated that this was a grade II AVM, which has a low surgical risk.^4^

The AVM classification by Nisson et al. indicates that this was a grade I AVM, which has a low risk assessment.^5^

Considering that flow-related aneurysms are distal and that, after treatment of the AVM, 80% of distal flow-related aneurysms disappear and that there was no hemorrhage in our case, we decided for conservative treatment of these aneurysms.^6^

This was an unruptured AVM with a high risk of bleeding and low surgical assessment risk. Therefore, we decided on interventional treatment with combined strategies with microsurgery after embolization.

**3:29 Embolization.** We proceeded to embolize the large arterial feeders, identified by the pink arrow, with Onyx to decrease the blood flow and facilitate surgical resection. The yellow arrow indicates the cortical segment of the PICA that was not embolized.

This video shows the result of the embolization of the terminal branches of the PICA. After the procedure, the basilar artery was filled.

**4:02 Positioning.** The patient was in a three-quarter prone position with mild flexion and lateral rotation of the head. The jugular veins were made straight to prevent problems of venous return and cerebellar congestion. The advantage of this position is that the abdomen is entirely free, so there is no risk of increased intraabdominal pressure, which also avoids venous return problems. A left occipital EVD was placed.

**4:32 Exposure.** An incision was made from the inion until the spinal process of C2.

The muscular layer was opened in a Y shape. Here, we show the occipital bone exposed. The blue dot is the inion. The red dot is the squamous part of the occipital bone. The orange dot is the posterior atlantooccipital membrane. The green dot is the posterior arch of C1.

A suboccipital craniotomy was performed, and the dura mater was opened. The blue arrow shows the foramen magnum.

**5:09** The arachnoid of the cisterna magna was opened, the main drainage vein of the AVM was viewed after the opening of the arachnoid membrane, and a corticectomy was performed. The lateral edge of the AVM was isolated to avoid creating a fox hole.

The deep arterial feeders from the left PICA are shown here. These arteries are clipped to promote a decrease in the blood supply to the AVM. This helps to facilitate the dissection of the AVM and to avoid bleeding in the surgical field. The embolized vessels can be seen here.

**5:49** Then, these clipped arteries are cut, and the telovelotonsillar segment of the PICA can be seen preserved here.

**5:57** The drainage vein was identified, and after the complete removal of the arterial feeders, the vein started to shrink, and there was no more blood return to the vein. Then, the vein is clipped. After clipping, there was no blood flow to the AVM. When this was confirmed, the drainage vein was cut.

We proceeded to hemostasis of the surgical field.

**6:27** This drawing shows the surgical field after the removal of the AVM. The arrow shows the dura mater. This is where the AVM was located. The clipped arterialized drainage vein is here. The PICA is depicted transparently in this drawing. These are the clipped final branches of the telovelotonsillar segment of the PICA, and this is the cortical segment of the PICA that was preserved.

**6:59 Closure.** The dura mater was closed, and we used a flap of pericranium to perform the duraplasty. Then, we proceeded to the cranioplasty.

The muscular layer was closed with an anchored suture using a multifilament absorbable suture. The subcutaneous tissue and the skin were closed.

**7:21 Postoperative Imaging.** Here, postoperative arteriography of both the right and left vertebral arteries shows that the cerebellar AVM was completely removed. We observe that the cortical portion of the left PICA can still be seen.

**7:39 Postoperative Course.** The patient had no neurological deficits. The EVD was removed 2 days postoperatively. The patient was discharged home at postoperative day 6.

Regarding flow-related aneurysms, we initially follow up with CT angiography every 6 months.

## Author Contributions

Primary surgeon: Dellaretti. Editing and drafting the video and abstract: all authors. Critically revising the work: all authors. Reviewed submitted version of the work: all authors. Approved the final version of the work on behalf of all authors: Dellaretti. Supervision: Dellaretti.

## Supplemental Information

### Online-Only Content

Supplemental material is available online.

*Supplemental Fig. 1.* https://thejns.org/doi/suppl/10.3171/2020.10.FOCVID2058.
