# Supplementary material for: Combined strategies on the treatment of cerebellar arteriovenous malformation
Source: Neurosurg Focus Video. 2021 Jan 1;4(1):V16. doi: 10.3171/2020.10.FOCVID2058 (PMC9542499; doi:10.3171/2020.10.FOCVID2058)
Supplement: Supplementary file 2 [file SupplementalFig1_FOCVID20-58.pdf]

NEUROSURGICAL FOCUS VIDEO

ONLINE ONLY

Supplemental material

Combined strategies on the treatment of cerebellar arteriovenous malformation

Dellaretti et al.

https://thejns.org/doi/abs/10.3171/2020.10.FOCVID2058

DISCLAIMER The *Journal of Neurosurgery* acknowledges that the following section is published verbatim as submitted by the authors and did not go through either the *Journal's* peer-review or editing process.

© 2021 The authors, CC BY 4.0 (http://creativecommons.org/licenses/by/4.0/)

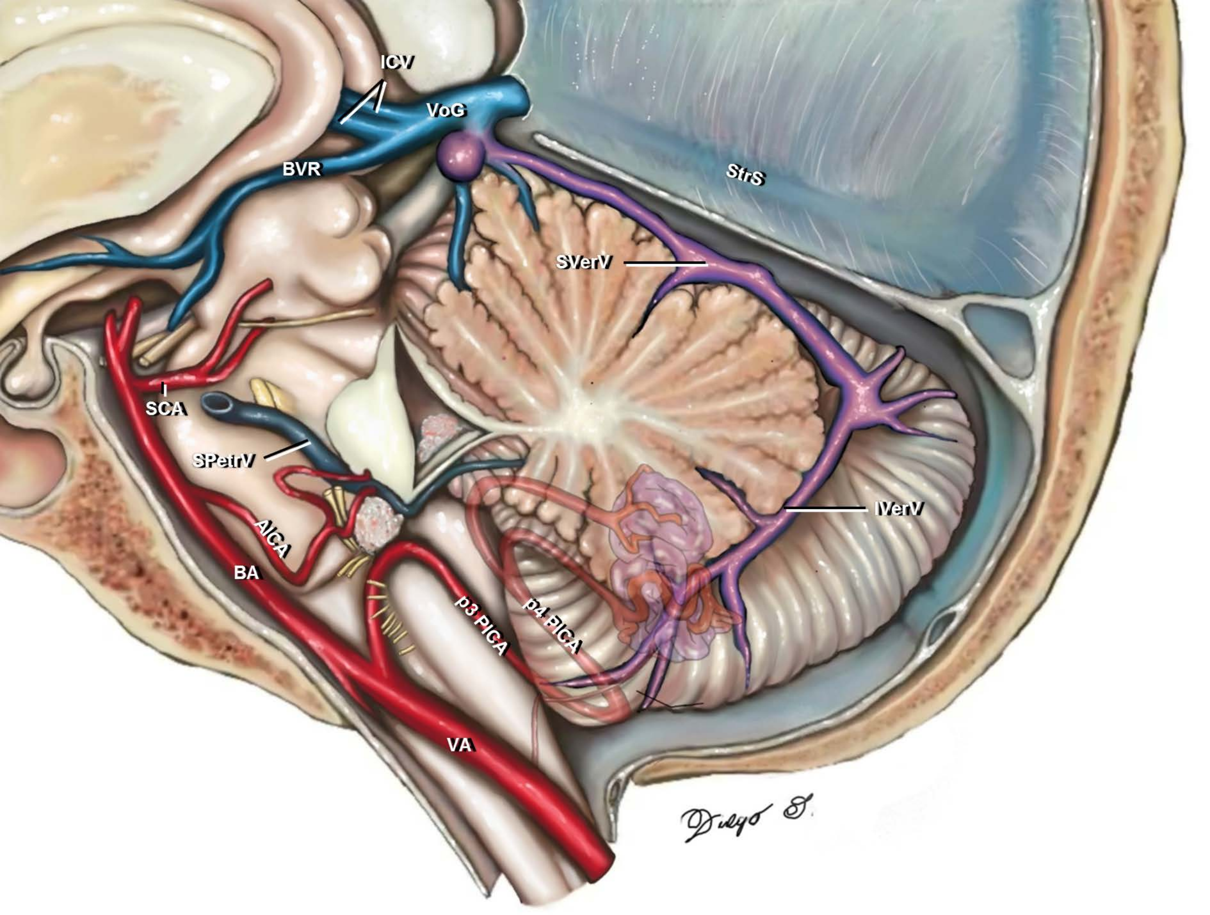

Supplemental Figure 1. Inferior Vermian AVM. Sagittal view. AICA = anterior inferior cerebellar artery; BA = basilar artery; BVR = basal vein of Rosenthal; ICV = internal cerebral vein; IVerV = inferior vermian vein; PICA = posterior inferior cerebellar artery; p3 PICA = PICA, tonsillomedullary segment; p4 PICA = PICA, telovelotonsillar segment; SCA = superior cerebellar artery; SPetrV = superior petrosal vein; StrS = straight sinus; SVerV = superior vermian vein.
